# Experimental *Lachesis muta rhombeata* envenomation and effects of soursop (*Annona muricata*) as natural antivenom

**DOI:** 10.1186/s40409-016-0067-6

**Published:** 2016-03-08

**Authors:** Caroline Marroni Cremonez, Flávia Pine Leite, Karla de Castro Figueiredo Bordon, Felipe Augusto Cerni, Iara Aimê Cardoso, Zita Maria de Oliveira Gregório, Rodrigo Cançado Gonçalves de Souza, Ana Maria de Souza, Eliane Candiani Arantes

**Affiliations:** Department of Physics and Chemistry, School of Pharmaceutical Sciences of Ribeirão Preto, University of São Paulo (USP), Ribeirão Preto, SP Brazil; Department of Clinical Analyses, Toxicology and Food Sciences, School of Pharmaceutical Sciences of Ribeirão Preto, University of São Paulo (USP), Ribeirão Preto, SP Brazil; Núcleo Serra Grande for Captive Breeding of Lachesis muta rhombeata, CTF Ibama MMA, 495100 Itacaré, BA Brazil

**Keywords:** *Lachesis muta rhombeata*, Bushmaster, Natural antivenom, Antiophidic action, Soursop, *Annona muricata* L.

## Abstract

**Background:**

In the Atlantic forest of the North and Northeast regions of Brazil, local population often uses the fruit juice and the aqueous extract of leaves of soursop (Annona muricata L.) to treat *Lachesis muta rhombeata* envenomation. Envenomation is a relevant health issue in these areas, especially due to its severity and because the production and distribution of antivenom is limited in these regions. The aim of the present study was to evaluate the relevance of the use of soursop leaf extract and its juice against envenomation by *Lachesis muta rhombeata*.

**Methods:**

We evaluated the biochemical, hematological and hemostatic parameters, the blood pressure, the inflammation process and the lethality induced by *Lachesis muta rhombeata* snake venom. We also assessed the action of the aqueous extract of leaves (AmL) and juice (AmJ) from *A. muricata* on the animal organism injected with *L. m. rhombeata* venom (LmrV) in the laboratory environment.

**Results:**

LmrV induced a decrease of total protein, albumin and glucose; and increase of creatine kinase, aspartate aminotransferase, and urea concentrations. It provoked hemoconcentration followed by reduction of hematocrit, an increase in prothrombin time and partial thromboplastin time and a decrease of the blood pressure. LmrV induced the release of interleukin-6, an increase in neutrophils and changes in the serum protein profile, characteristic of the acute inflammatory process. LD_50_ values were similar for the groups injected with LmrV and treated or untreated with AmJ and AmL*.* Both treatments play a role on the maintenance of blood glucose, urea and coagulation parameters and exert a protective action against the myotoxicity. However, they seem to worsen the hypotension caused by LmrV.

**Conclusion:**

The treatments with AmJ and AmL present some beneficial actions, but they might intensify some effects of the venom. Therefore, additional studies on *A. muricata* are necessary to enable its use as natural antivenom for bushmaster snakebite.

## Background

The species *L. muta* is divided into two subspecies: *L. muta muta* found in tropical forests of Colombia, Venezuela, Guyana, Suriname, Peru, Ecuador and Brazil, and *L. muta rhombeata* confined to certain areas of the rainforest of the Brazilian Atlantic region [1, 2]. *Lachesis muta rhombeata* was considered “endangered of extinction” in 1989 by the official list of the Brazilian Institute of Environment and Renewable Natural Resources (IBAMA), and currently is considered “vulnerable” by the International Union for the Conservation of Nature [3].

The envenomation caused by *Lachesis* genus represents 4.5 % of all registered snakebites in Brazil and is characterized by the so-called “*Lachesis* Syndrome” [4]. Within the first few minutes after the bite, the victim is affected by agonizing burning throbbing local pain and edema, followed by intense inflammation, bleeding disorders, clotting disorders, kidney malfunction, myotoxicity and autonomic syndrome evidenced by sweating, nausea, vomiting, abdominal cramps, diarrhea, hypotension and bradycardia [5–9].

The venom is rich in proteolytic enzymes responsible for severe local effects such as swelling, local inflammation and necrosis mainly due to the action of phospholipases A_2_ (PLA_2_) and metalloproteinases [7]. Hemorrhagic effects are attributed to alpha-fibrinogenases, active on the factor XIII of the coagulation cascade, and hemorrhagic metalloproteinases that provoke microvascular damage in the organism, which leads to internal bleeding [10–16]. This effect is enhanced by the action of thrombin-like serine proteinases and C-type lectins that respectively induce the consumption of fibrinogen and cause platelet aggregation and hemaglutination [7, 17–24]. Together, they provoke the disturbance in blood coagulation and collaborate with the hemorrhagic profile observed during the envenomation.

The PLA_2_ exerts indirect hemolytic effect and plays a major role in neurotoxic symptoms (stimulation of the autonomic nervous system) and causes vomiting, diarrhea, sweating, hyper salivation, bradycardia and hypotension in human victims [7, 23, 25–31]. Other components of the venom include L-amino acid oxidases, bradykinin potentiating peptides, cysteine-rich secretory proteins, C-type natriuretic peptides, nerve growth factors and hyaluronidases [7, 24, 32–36].

Up to the present, the only specific therapy available for snake envenomation is the serotherapy. Its efficiency is mainly related to the amount of venom injected and the time elapsed between the accident and the start of treatment [37]. Despite being the treatment of choice, it is limited to regions that have structured health centers and may provoke several side effects, which makes the search for additional and/or alternative treatments even more important [6, 34]. Moreover, the venom of *L. muta* has low immunogenic capacity, when compared with other venoms [38].

Plants popularly used to maintain or restore human health provide an important source of compounds able to directly assist in the treatment of accidents with venomous animals, or indirectly, as a complement to conventional antivenom therapy. The use of plant extracts, as an antidote against venoms is an old option for many communities that need rapid access to antivenom therapy.

In the Atlantic forest of the North and Northeast regions of Brazil, the fruit juice and the aqueous extract of soursop (*Annona muricata* L.) leaves are often used by local population to treat *Lachesis muta rhombeata* envenomation [39]. The envenomation caused by *Lachesis* genus comprises an important health issue, especially in regions where the production and distribution of antivenom are limited. The search for alternative methods to minimize or delay the action of the venom is a necessity and should be encouraged.

The aim of the current study was to evaluate the relevance of the use of leaf extracts and juice of *Annona muricata* L., a plant traditionally used in the Brazilian Northeast region against *Lachesis muta rhombeata* venom.

## Methods

### Venom

The venom was kindly provided by Rodrigo C. Gonçalves de Souza, founder and director of the Núcleo Serra Grande for Captive Breeding of *Lachesis muta rhombeata,* Federal Technical Registry No. 495100. The present study was approved by IBAMA (process no. 14785–1).

### *Annona muricata* L.

The aqueous extract of *Annona muricata* L. leaves (AmL) was prepared by maceration of 20 g of fresh leaves newly collected in the presence of 80 mL of water and then the extract was filtered through a clean cotton fabric. *Annona muricata* L. juice (AmJ) was prepared in proportion of 50 % pulp and 50 % drinking water. The leaf extract and juice were administered by gavage to animals. For this study, three preparations of 0.5 mL (water, AmJ or AmL) were administered by gavage, 1 h and 15 min before and 1 h after intramuscular (i.m.) injection of saline (control, 200 μL) or venom (200 μL, 3 mg/rat). The voucher specimen is deposited at the Herbarium of University of São Paulo – SPFR, in the city of Ribeirão Preto, SP, Brazil, under the collector identification MGroppo 1886.

### Experimental groups

Animal care was in accordance with ethical recommendations of the International Guiding Principles for Biomedical Research Involving Animals and the study was approved by the Ethics Commission for the Use of Animals (CEUA) of the University of São Paulo, Campus of Ribeirão Preto (process no 07.1.277.53.1). Male Wistar rats (209 ± 12 g) were supplied by the Central Biotherium (University of São Paulo, Campus of Ribeirão Preto).

The animals were divided into six groups that were analyzed in different times. Each animal from the groups LmrV, LmrV + AmL and LmrV + AmJ received an intramuscular injection of 3 mg of venom diluted in physiological solution (200 μL), which is a previously determined non-lethal dose (data not shown) and received, respectively, water, aqueous extract of soursop leaves and soursop juice. The control groups C, C + AmL and C + AmJ received an intramuscular injection of physiological solution (200 μL) followed by water, aqueous extract of soursop leaves and soursop juice, respectively. Each group was evaluated in three different times after injection of physiological solution or venom: 1, 6 and 24 h.

At the appointed time for each subgroup (1, 6 or 24 h after injection), the animals were anesthetized with a intraperitoneal injection of a mixture of ketamine (35 mg/kg) and xylazine (5 mg/kg), and subjected to cardiac puncture for collecting blood. Then, they were euthanized in a CO_2_ chamber.

### Biochemical parameters

The concentrations of albumin, total protein, blood glucose, urea, creatinin, aspartate aminotranspherase (AST) and creatine kinase (CK) were evaluated in the serum of the animals submitted to different treatments. All biochemical analyses were performed at the Laboratory of Clinical Analysis of the School of Pharmaceutical Sciences of Ribeirão Preto in an automated analyzer BT 3000 Plus (Wiener Lab, Argentina, serial number 41080340).

### Hematological parameters

Hematological parameters were determined in whole blood of animals using classical methods: volume of packed red blood cells or hematocrit (Ht) by the microhematocrit method, total hemoglobin concentration (Hb) by the cyanmethemoglobin method, red blood cells (RBC) count with a Neubauer hemacytometer using Hayem diluting fluid.

### Hemostatic parameters

The prothrombin time and partial thromboplastin time were determined using a commercial kit of Wiener Lab (Wiener Lab, Argentina).

### Blood pressure

The blood pressure was evaluated by tail-cuff plethysmography with heating [40, 41].

### Inflammatory process

The assays used to evaluate the inflammatory profile triggered by *Lachesis muta rhombeata* envenomation were the global leukocyte count, differential leukocyte count, serum protein quantification after electrophoresis in agarose gel and concentration of interleukin-6, determined by enzyme immunoassay (commercial kit from R & D Systems). Serum proteins were separated by agarose electrophoresis [42]. Bands were quantified by densitometry (DenGo Densitometer, Qualiterm, Brazil).

### Lethality assay

The lethal dose 50 (LD_50_) for *Lachesis muta rhombeata* venom was calculated by the probits method [43]. Three experimental groups (see below) of male Balb-C mice (22 ± 3 g) were injected with four different doses of venom (100 μL, i.m.): 10, 20, 40 and 80 mg/kg (ten animals per dose). The choice of the venom doses was based on the studies of Otero et al. [25].

1. LmrV: animals received water (150 mL) by gavage 15 min before venom injection.
2. LmrV + AmL: animals were administered aqueous extract of soursop leaves (150 mL) by gavage 15 min before venom injection.
3. LmrV + AmJ: animals received soursop juice (150 mL) by gavage 15 min before venom injection.

### Statistical analysis

The results are expressed as mean ± standard error of mean (SEM). The statistical analysis was carried out by one-way ANOVA followed by Tukey-Kramer post-hoc test with a significance level set at *p* < 0.05, using Graphpad Prism® 4.0 software for Windows™.

## Results and discussion

### Biochemical parameters

**Fig. 1:**
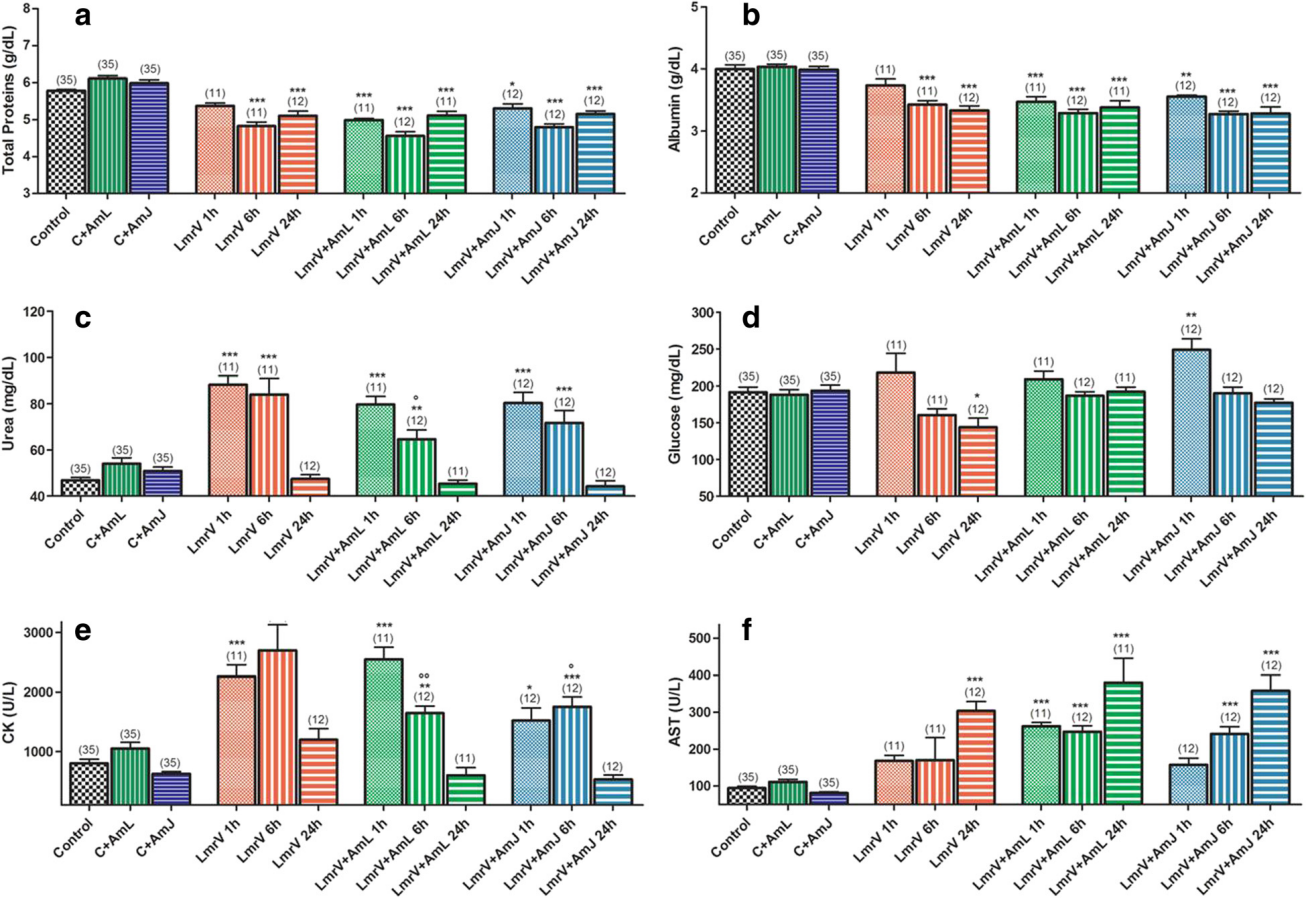
**a** Concentration of total proteins, **b** albumin, **c** urea, **d** glucose, **e** CK and **f** AST in the serum of male Wistar rats injected with 3 mg of *Lachesis muta rhombeata* venom, at 1, 6 and 24 h after envenomation (LmrV) and with their respective treatments with aqueous extract of *Annona muricata* L. leaves (LmrV + AmL) or *Annona muricata* L. juice (LmrV + AmJ). The numbers above the columns represent the number of animals used in each test. *** *p* < 0.001, ** *p* < 0.01, * *p* 0.05 compared to control group. *p* < 0.05, °° *p* < 0.01 compared with the envenomation group (LmrV)

A time-dependent decrease is observed for total protein and albumin (Fig. 1 – a and b) in groups in which animals were injected with venom, which is consistent with the intensity of the inflammatory process triggered by the envenomation. The biochemical changes observed in *L. muta rhombeata* envenomation are consequences of the main action of enzymes present in the venom, such as PLA_2_, thrombin like serine proteinases and metalloproteinases. PLA_2_ and metalloproteinases exert intense inflammatory and myotoxic effects. The inflammatory process triggers an acute phase reaction, altering the concentration of serum proteins, mainly albumin, which is part of the group of negative acute phase inflammatory proteins, and its synthesis is reduced during the inflammatory process. The decrease of total protein concentration occurs along with the reduction of albumin concentration, since this is the most abundant protein in blood [44].

The results show an increase of serum urea (Fig. 1 – c) during the early hours after the venom injection in the untreated group (LmrV 1 h and LmrV 6 h) and in treated groups (LmrV + AmL, 1 h and 6 h; LmrV + AmJ, 1 h and 6 h), but increases in serum urea observed in the treated groups were lower (6 h) than those observed in untreated animals, showing that treatment may be able to partially reverse this effect of the venom. After 24 h of envenomation, the concentration of urea tend to return to control values.

The increased concentrations of urea and creatinine are commonly observed after *Lachesis muta* envenomation in humans [6, 45]. The fibrin clots may be responsible for this response observed in the groups injected with venom. According to Souza [45] and Lima et al. [23], the increase of urea is possibly the result of deposition of fibrin clots in the kidneys due to the action of thrombin-like serine proteinases present in *Lachesis* venom, which promotes thrombin cleavage into fibrinogen and fibrin clots. The venom-induced renal damage could also be a consequence of the myoglobin deposit in the kidney, since the venom is myotoxic, or due to periods of renal hypotension [29, 46].

The increase in creatinine concentration is usually accompanied by increased concentrations of urea, as indicative of decreased glomerular filtration rate. Pardal et al. [8] in a case report, noted an increase in creatinine during the first day after the snakebite, which returned to normal levels of concentration after that. Although the animals had shown increased urea levels in the early hours (1 and 6 h) after the envenomation, no changes were observed in serum creatinine (data not shown).

A time-dependent decrease was also observed in blood glucose (Fig. 1 – d) in the group injected with LmrV as well as the maintenance of normal glucose values in the group treated with aqueous extract of *Annona muricata* L. leaves (LmrV + AmL), evidencing that AmL treatment was able to control glucose levels. Taylor [47] describes the popular use of soursop to control the blood glucose levels of diabetic people, in Amazonian region of Peru.

The Fig. 1 – E shows that LmrV induces an increase of CK, which is a biomarker of muscle damage, in the 1^st^ and 6^th^ hours, while Fig. 1 – F shows an increase in AST mainly 24 h after envenomation. These effects are probably due to the myotoxic action of venom PLA_2_, as well as the action of metalloproteinases that indirectly lead to death of muscle fibers, due to the reduction of tissue perfusion [23, 26, 29,]. CK levels in groups LmrV + AmL and LmrV + AmJ, both 6 h after envenomation, are lower than that observed in the untreated group LmrV (Fig. 1 – e), suggesting a protective effect of the juice and aqueous extract of soursop leaves against myotoxic activity of the venom. Although not significant, there is a reduction of CK concentration 24 h after envenomation in the treated groups when compared to the untreated group (LmrV 24 h), which reinforces the hypothesis that the treatment may have protective effect against the myotoxicity of *Lachesis muta rhombeata* venom.

There was an increase in AST (Fig. 1 – f) observed in groups LmrV, LmrV + AmL and LmrV + AmJ, mainly 24 h after injection of the venom. AST is a sensitive marker for liver damage, but its significant increase in groups injected with LmrV does not necessarily indicates liver damage, since AST is also distributed in appreciable amounts in cardiac tissues, muscles, red blood cells, brain and kidney, and this increase may be due to hemolysis and/or myotoxicity caused by the envenomation [48]. In case reports published by Jorge et al. [7] and Torres et al. [49], both also described increased liver transaminases after *Lachesis muta* envenomation in humans.

### Hematological parameters

**Fig. 2:**
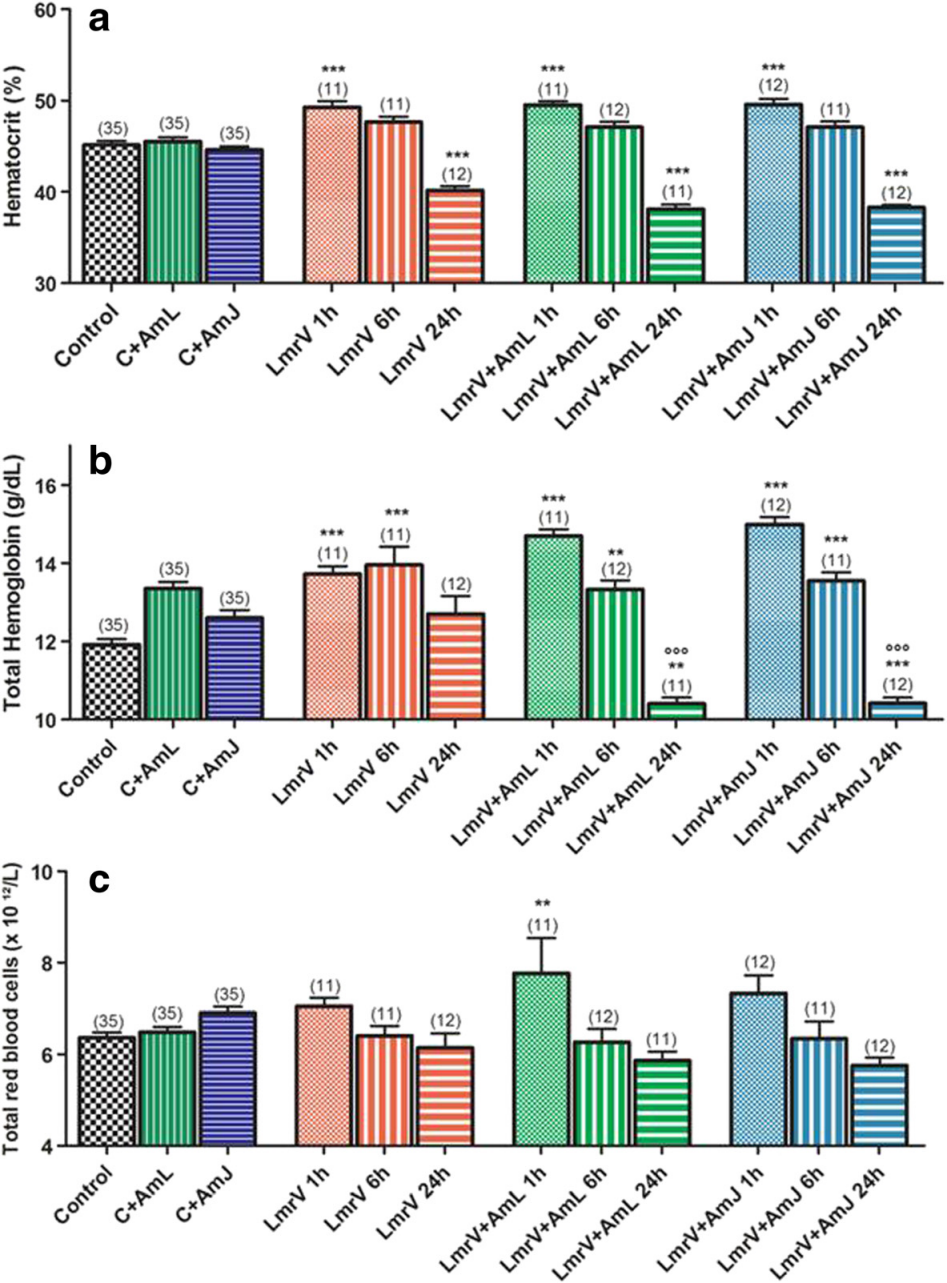
**a** Hematocrit, **b** total hemoglobin concentration, **c** total red blood cells count in whole blood of male Wistar rats injected with 3 mg of *Lachesis muta rhombeata* venom, at 1, 6 and 24 h after envenomation (LmrV) and with their respective treatments with aqueous extract of *Annona muricata* L. leaves (LmrV + AmL) or *Annona muricata* L. juice (LmrV + AmJ). The numbers above the columns represent the number of animals used in each test. *** *p* < 0.001, ** *p* < 0.01 compared to control group. °°° *p* < 0.001 compared to the envenomation group (LmV)

Our results showed a significant initial increase (1 h) of hematocrit values followed by decay (24 h), in the groups injected with venom (Fig. 2 – a), with and without treatment (LmrV, LmrV + AmL and LmrV + AmJ). These values drop significantly after 24 h. The same effect, an initial increase and decrease after 24 h, was also observed in total hemoglobin concentrations (Fig. 2 – b), as well as in the red blood cell count (Fig. 2 – c), being the increase significant only in the group LmrV + AmL (1 h).

The treatments with AmL and AmJ appear to worsen the hematological profile induced by LmrV, since the treated groups showed a significant decrease of hemoglobin (24 h) compared with LmrV group (Fig. 2 – b).

An evidence of envenomation by *Lachesis muta rhombeata* is indirect hemolytic effect induced by the PLA_2_ present in the venom, which acts on the phospholipids of the cell membrane, causing the lysis of red blood cells [25, 45]. Campos et al. [50] also attributed the hemolytic effect to PLA_2_, since inhibition of this toxin by triazole derivatives is capable of neutralizing the hemolytic activity of *Lachesis muta* venom. This effect probably occurs due to the initial hemoconcentration caused by plasma extravasations to tissues and dehydration due to diarrhea, probably due to the hemolytic action of the venom toxins [25, 45, 51]. One hypothesis to justify this result with the treated groups is the effect of soursop to treat diarrhea, preventing the fluid loss by the animal [47]. In this sense, for the same amount of hemoglobin, there is a greater volume of plasma, leading to decreases in the total hemoglobin concentration. The same pattern is observed in the red blood cell count and hematocrit (Fig. 2 – a and c).

Several case reports show hematological changes during the envenomation. Pardal et al. [8] reported these changes during 24 h after envenomation by *Lachesis*. On admission at the hospital, the hematocrit of the patient was 40 % and the total hemoglobin 11.3 g/L. After 24 h, the hematocrit was under 25 % and hemoglobin 8.1 g/L. A similar profile was described by Jorge et al. [7], in which they report hematocrit values of 54 % two hours after the snake bite, and 47 % after 24 h. The same occurred with the hemoglobin concentration, which was 18.2 g/dL after two hours and 16 g/dL after 24 h of envenomation. The decrease of hemoglobin concentration was accompanied by reduction of total red blood cells.

### Hemostatic parameters

**Fig. 3:**
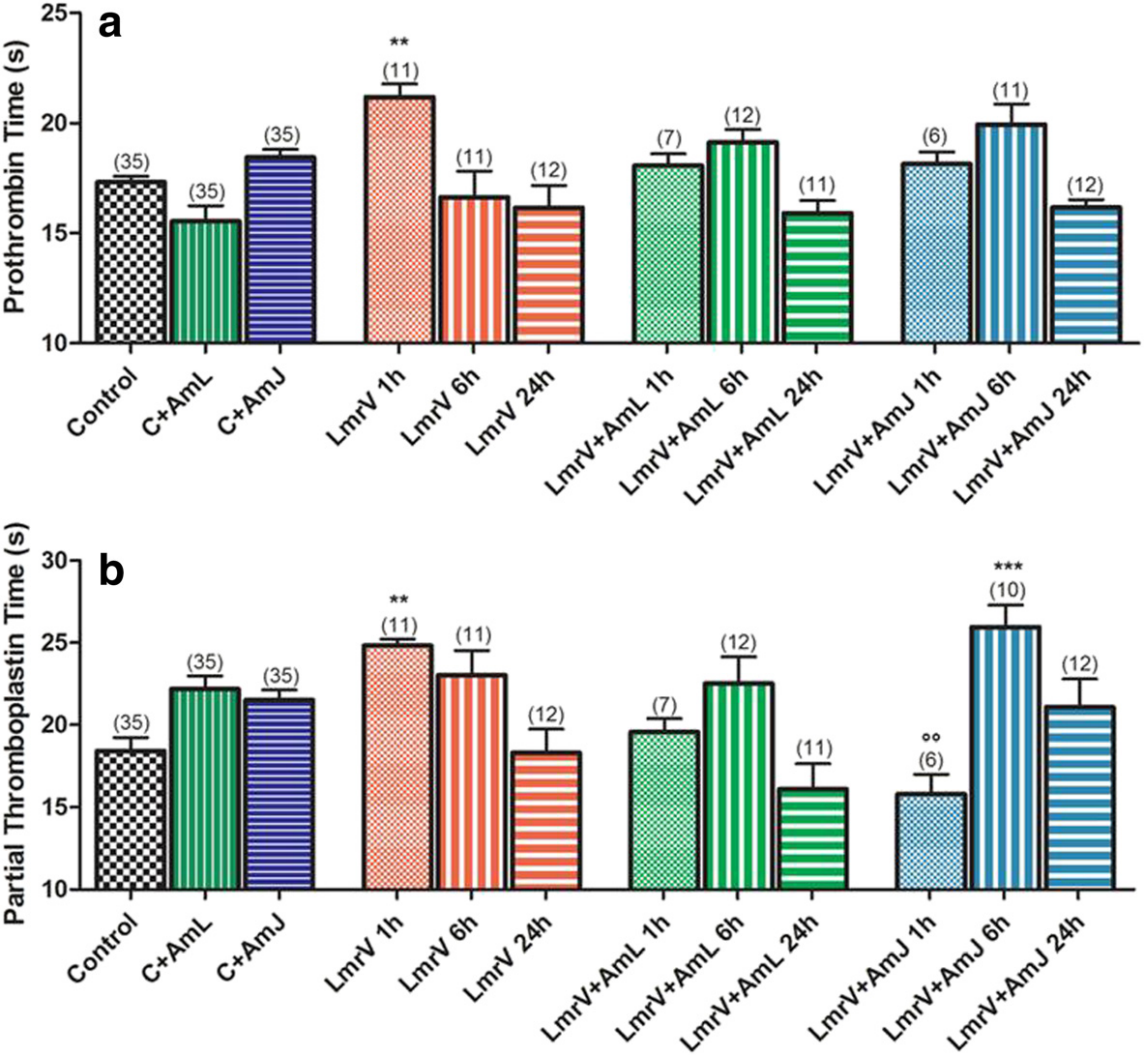
**a** Determination of prothrombin time and **b** partial thromboplastin time in plasma of male Wistar rats injected with 3 mg of *Lachesis muta rhombeata* venom, at 1, 6 and 24 h after envenomation (LmrV) and with their respective treatments with aqueous extract of *Annona muricata* L. leaves (LmrV + AmL) or *Annona muricata* L. juice (LmrV + AmJ). The numbers above the columns represent the number of animals used in each test. *** *p* < 0.001, ** *p* < 0.01 compared to control group. °° *p* < 0.001 compared to the envenomation group (LmrV)

We found a significant increase in prothrombin time (Fig. 3 – a) and an increase of partial thromboplastin time (Fig. 3 – b) in the first hour after the venom injection (LmrV 1 h). A tendency to normalization was observed in the following hours (6 and 24 h). Groups treated with AmL and AmJ maintained prothrombin time at values close to the control group, suggesting a positive effect of the treatments with *Annona muricata* L. on hemostasis. In the case reports published by Pardal et al. [8] and Jorge et al. [7] there are descriptions of patients with incoagulable blood (clotting time test) even 26 h after the snakebite, and increased prothrombin time and partial thromboplastin time values two hours after the envenomation.

The envenomation by snakes of the Viperidae family induces changes in hemostasis and causes hemorrhagic syndromes. These processes involve a large number of molecules that promote microvascular damage or interfere with the coagulation cascade, and as pro- and antiaggregating toxins affecting blood platelets. Metalloproteinases, thrombin-like serine proteinases, PLA_2_ and type-C lectins are involved in this process [10, 17, 23, 27, 52–55]. Together they cause an imbalance in the hemostatic system and tissues repair, leading to persistent bleeding.

### Blood pressure

The evaluation of blood pressure during *Lachesis* envenomation is very important, since the snakebite causes an intense decrease in blood pressure, commonly seen in envenomation in humans [45]. The hypotension is provoked by the combined autonomic and hemorrhagic effects as well as bradycardia. The vagal symptomatology consists of the following symptoms: sweating, nausea, vomiting, abdominal cramps, diarrhea, hypotension and bradycardia and is probably caused by PLA_2_ [29, 56]. The hemorrhagic effects are due to the actions of thrombin-like serine proteinases and metalloproteinases, which deplete the reserves of fibrinogen and cause microvascular damage, respectively.

**Fig. 4:**
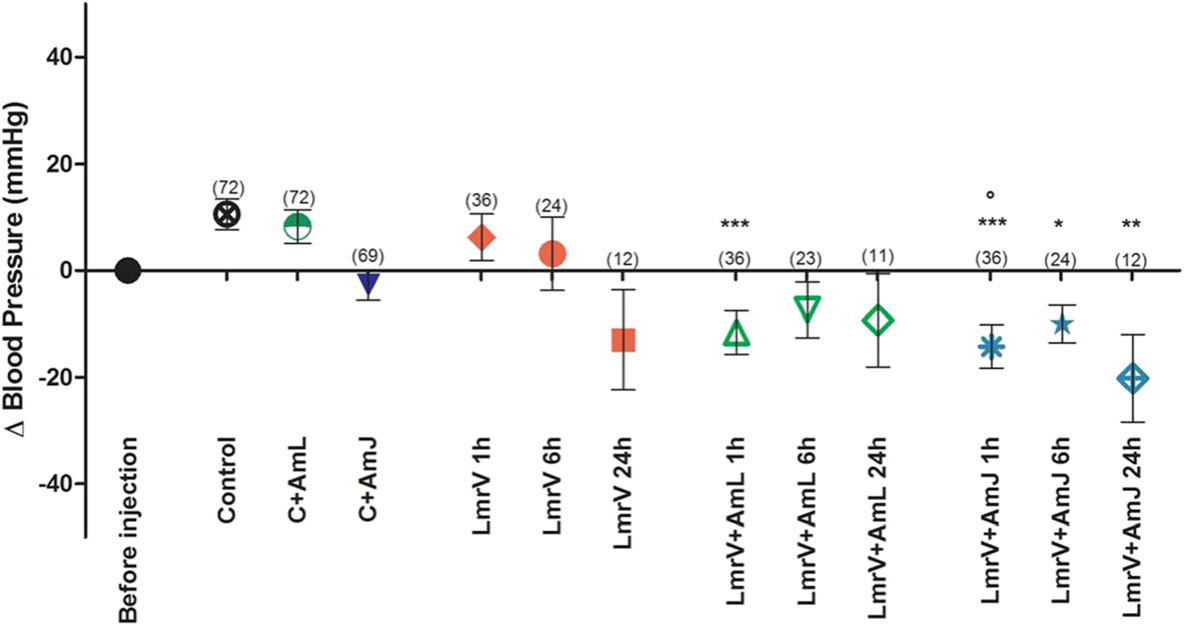
Variation of systolic blood pressure during 24 h after the envenomation with *Lachesis muta rhombeata* venom (LmrV) and their respective treatments with aqueous extract of *Annona muricata* L. leaves (LmrV + AmL) or *Annona muricata* L. juice (LmrV + AmJ). The animals, male Wistar rats, were injected with 3 mg of *Lachesis muta rhombeata* venom and the blood pressure was measured before the venom injection and at 1, 6 and 24 h after the envenomation. The numbers above the columns represent the number of animals used in each test

A time-dependent tendency of blood pressure reduction was observed in the animals injected with LmrV, especially after 24 h, in all groups analyzed (Fig. 4). It is important to note that the reduction of blood pressure was more pronounced in animals injected with LmrV and treated with AmL or AmJ than in animals only injected with LmrV, especially 1 and 6 h after envenomation. Although the treatment with the juice (LmrV + AmJ) has not presented significant differences when compared to the untreated group (LmrV), the results indicate that AmJ may potentiate the hypotensive effect of LmrV. We also noted a slight hypotensive effect in the control group of *Annona muricata* L. juice (C + AmJ).

Studies in animal models demonstrated hypotensive, vasodilator and cardiac depressant effects of *Annona muricata* L. and its use is not recommended for people with hypotension [47]. Due to this natural effect of the plant, the treatment with soursop juice could intensify the hypotension observed in human envenomation, as shown in case reports of snakebites caused by *Lachesis* genus [9, 45].

*A. muricata* L. reduces blood pressure by a mechanism that does not involve muscarinic, endothelial, histaminergic, adrenergic or endothelial-dependent pathways. The more likely mechanism of action is Ca^2+^ antagonism, involving voltage dependent Ca^2+^ channel blockade and/or inhibition of Ca^2+^ release from intracellular stores of the blood vessels [57]. The hypotensive effect of *A. muricata* may be attributed to the combined action of alkaloids and essential oils present in the plant. The alkaloids, isoquinoline, coreximine and anomurine exert a transient depressive effect on the blood pressure. The essential oil beta-caryophyllene exhibits hypotensive and vasodilator effects. Furthermore, reticuline, another alkaloid found in *Annona muricata* leaves, can cause hypotension through voltage-dependent Ca^2+^ channel blockade and/or inhibition of Ca^2+^ release from norepinephrine-sensitive intracellular stores [57].

### Inflammatory process

#### Serum proteins

Most of serum proteins are involved in the inflammatory response, as positive acute-phase proteins or negative acute-phase proteins [42]. Our analysis (Table 1) shows the serum protein profile of animals at different times (1, 6 and 24 h) after envenomation (LmrV) and treated with AmL or AmJ, showing alterations related to the inflammatory response induced by LmrV. The albumin significantly decreases 6 and 24 h after LmrV injection, with and without the treatments (AmL and AmJ), a result that is confirmed by the biochemical analysis of this protein (Fig. 1 – b).

**Table 1:** Percentages of serum proteins of the animals at different times after envenomation

Groups	*N* ^a^	Albumin (%)^b^	Alfa-1 (%)^b^	Alfa-2 (%)^b^	Beta-1 (%)^b^	Beta-2 (%)^b^	Gama (%)^b^
Control	36	59.84 ± 0.51	15.10 ± 0.72	8.61 ± 0.31	5.76 ± 0.19	8.39 ± 0.18	2.02 ± 0.08
C + AmL	27	59.46 ± 0.67	14.88 ± 0.63	9.67 ± 0.62	5.84 ± 0.19	8.28 ± 0.17	1.92 ± 0.10
C + AmJ	28	59.66 ± 0.66	15.61 ± 0.86	6.60 ± 0.62	6.20 ± 0.23	8.88 ± 0.18	2.32 ± 0.13
LmrV 1h	10	56.14 ± 1.24	18.72 ± 1.17	8.15 ± 0.72	5.69 ± 0.20	8.76 ± 0.31	2.54 ± 0.27
LmrV 6h	10	55.56 ± 0.81*	18.20 ± 0.75	9.47 ± 0.74	6.52 ± 0.43	8.03 ± 0.26	2.21 ± 0.24
LmrV 24h	9	45.60 ± 0.92*	21.22 ± 0.78*	14.82 ± 1.90*	12.33 ± 0.28*	8.80 ± 0.24	1.70 ± 0.27
LmrV + AmL 1h	5	56.50 ± 0.54	20.44 ± 0.45	6.34 ± 0.26	5.76 ± 0.22	9.12 ± 0.22	1.62 ± 0.20
LmrV + AmL 6h	6	54.20 ± 0.41*	19.30 ± 0.52	7.68 ± 0.60	7.25 ± 0.20	9.50 ± 0.36	1.70 ± 0.27
LmrV + AmL 24h	5	40.70 ± 1.81*	17.10 ± 1.36	12.82 ± 0.44*	10.42 ± 0.30*	15.28 ± 2.56*°	2.12 ± 0.24
LmrV + AmJ 1h	6	57.43 ± 0.73	16.55 ± 0.34	9.82 ± 0.58	5.53 ± 0.33	8.50 ± 0.34	2.16 ± 0.14
LmrV + AmJ 6h	6	52.78 ± 1.48*	20.02 ± 0.66	7.28 ± 0.49	6.98 ± 0.68	9.97 ± 0.26	2.66 ± 0.42
LmrV + AmJ 24h	6	40.33 ± 1.81*	19.35 ± 1.01	10.95 ± 0.99	10.87 ± 0.44*	14.70 ± 2.22*°	2.05 ± 0.24

Male Wistar rats were injected with 3 mg of *Lachesis muta rhombeata* venom, at 1, 6 and 24 h after envenomation (LmrV) and treated respectively with aqueous extract of *Annona muricata* L. leaves (LmrV + AmL) or *Annona muricata* L. juice (LmrV + AmJ)

^a^Number of animals per group. ^b^Percentages of serum proteins estimated by densitometry, represented by mean ± SEM. * *p* < 0.05, significant difference compared to control group. ***°*** *p* < 0.05, significant difference compared to envenomation LmrV group (without treatment)

The alpha-1 zone is composed of positive acute-phase proteins such as proteinase inhibitors, and is increased 24 h after the envenomation without treatment (LmrV 24 h), due to the acute inflammatory process triggered by the venom. The same occurs with the alpha-2 zone, which is also composed of positive acute-phase proteins, and is increased in untreated groups (LmrV 24 h) and in the group treated with aqueous extract of *Annona muricata* L. leaves (LmrV + AmL 24 h).

The beta zone is mainly represented by transferrin and C3 component of complement system, and can be divided into beta-1 and beta-2 zones. There is a significant increase in beta zone in the groups injected with venom after 24 h, with and without treatment (LmrV 24 h, LmrV + AmL 24 h and LmrV + AmJ 24 h). The gama zone, composed by imunoglobulins, did not show any changes during the envenomation. These results confirm the inflammatory process triggered by the venom. Additionally, they show that treatments did not interfere with these parameters.

#### Interleukin-6

**Fig. 5:**
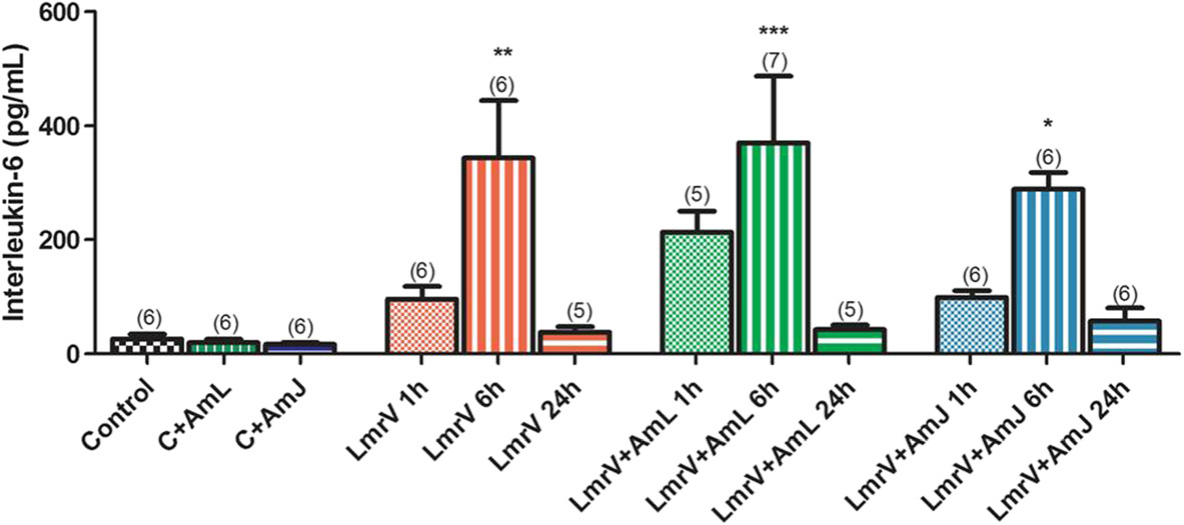
Interleukin-6 concentration of male Wistar rats injected with 3 mg of *Lachesis muta rhombeata* venom, at 1, 6 and 24 h after envenomation (LmrV) and with their respective treatment with aqueous extract of *Annona muricata* L. leaves (LmrV + AmL) or *Annona muricata* L. juice (LmrV + AmJ). The numbers above the columns represent the number of animals used in each test. *** *p* < 0.001, ** *p* < 0.01, * *p* < 0.05 compared to control group

We observed increased concentrations of IL-6 mainly 6 h after envenomation (Fig. 5). Treatment did not interfere with this LmrV effect. The IL-6 increase is consistent with other results already obtained, initiating and propagating the acute phase inflammatory response to the injury induced by the envenomation.

IL-6 has both pro-inflammatory and anti-inflammatory properties. It is often used as a marker to evaluate the inflammatory process and has been correlated with the severity of envenomation, together with other pro-inflammatory cytokines, such as TNF-alpha, IL-1b, IL-8, IL-10, IL-12 and COX-2 [58–63]. IL-6 cannot be uniquely related to pro-inflammatory response. It is known that the soluble form of IL-6 receptor is related to its pro-inflammatory activity whereas classic IL-6 is required for regenerative or anti-inflammatory activities of the cytokine [64, 65].

#### Total and relative white blood cells count

**Fig. 6:**
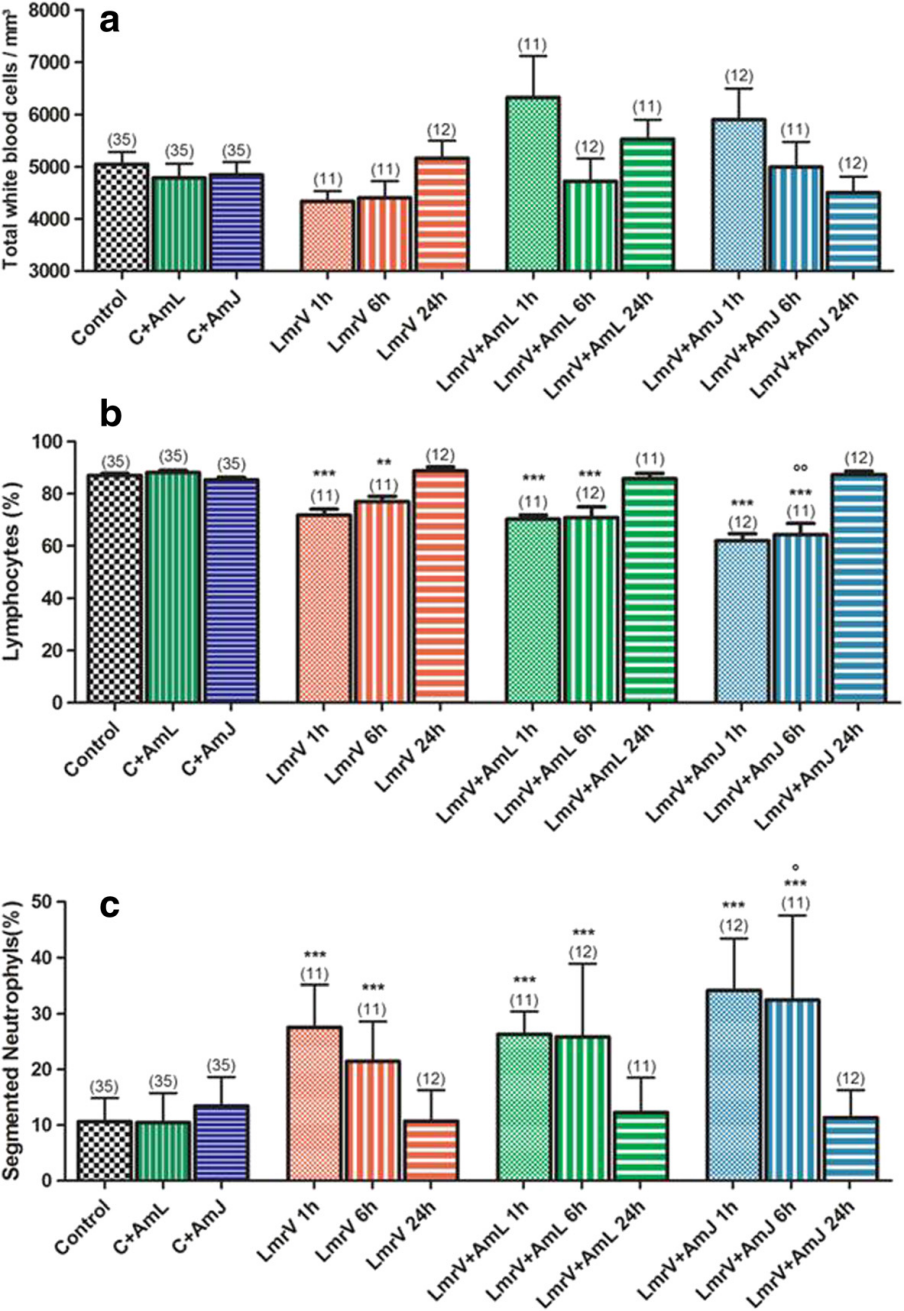
**a** Total white blood cells count, **b** relative count of lymphocytes and **c** segmented neutrophils of male Wistar rats injected with 3 mg of *Lachesis muta rhombeata* venom, at 1, 6 and 24 h after envenomation (LmrV) and with their respective treatments with aqueous extract of *Annona muricata* L. leaves (LmrV + AmL) or *Annona muricata* L. juice (LmrV + AmJ). The numbers above the columns represent the number of animals used in each test. *** *p* < 0.001, ** *p* < 0.01 compared to control group. °° *p* < 0.001, ° *p* < 0.001 compared to envenomation group without treatment (LmV)

The venom itself was not sufficient to alter significantly the total white blood cell count (Fig. 6 – a). However, there was a time-dependent trend in the increasing number of leukocytes in animals injected with venom without treatment (LmrV).

The differential leukocyte count of the groups injected with *Lachesis muta rhombeata* venom clearly shows the reduction of lymphocytes in the early hours after envenomation over the increasing of neutrophil number (Fig. 6 – b and c, respectively), and an increase of lymphocytes 24 h after envenomation accompanied by a decrease in the number of neutrophils. This effect of the venom on defense cells can be explained by the clonal expansion of B lymphocytes during the production of antibodies specific for the toxins present in the venom [66]. Neutrophilia occurs in the early hours of envenomation and returns to normal within 24 h after envenomation, indicating the recruitment of these cells to sites where tissue damages were intense.

### Lethality assay

The determination of the lethal dose 50 % (LD_50_) is essential for the standardization of antivenoms of natural origin, which justifies this test in order to evaluate the efficiency of treatment with AmL and AmJ for cases of *Lachesis muta rhombeata* envenomation*.*

The LD_50_ obtained in this study in the untreated group (LmrV) was 51.0 ± 6.4 mg/kg (44.6 to 57.4 mg/kg). The LD_50_ obtained for the group injected with the venom and treated with soursop juice (LmrV + AmJ) was 56.3 ± 8.5 mg/kg (47.8 to 64.8 mg/kg) and the LD_50_ obtained for the group injected with the venom and treated with aqueous extract of soursop leaves (LmrV + AmL) was 62.2 ± 6.8 mg/kg (55.4 to 69.0 mg/kg). These results show that the treatment did not significantly alter the LD_50_ values of the venom, when considering the fiducial limits for each test.

## Conclusion

The clinical profile of the envenomation caused by *Lachesis muta rhombeata* was well documented and very detailed for the first 24 h after the injection of the venom, and could evaluate the efficiency of the popular use of soursop (*Annona muricata* L.) as natural antivenom in snakebites caused by this specie.

In general, the treatments with AmL and AmJ do not alter relevantly the lethality of the envenomation, as the LD_50_ values have shown. They played a role in the maintenance of blood glucose and the coagulation parameters and exert a protective action against the myotoxicity of the venom. However, both treatments seem to worsen the hypotensive effect induced by LmrV. Therefore, additional studies with *A. muricata* are necessary to determine its suitable forms of use and mechanism of action in order to enable its safe and effective use as natural antivenom for bushmaster snakebite.

## Ethics approval

Animal care was in accordance with the ethical recommendations of the International Guiding Principles for Biomedical Research Involving Animals. The present study was approved by the Ethics Commission for the Use of Animals (CEUA) of the University of São Paulo, Campus of Ribeirão Preto (process no. 07.1.277.53.1). The use of snake venom was approved by IBAMA (process no. 14785–1).

## Abbreviations

AmJ: *Annona muricata* L. juice
AmL: *Annona muricata* L. leaves
AST: aspartate amino transferase
CK: creatine kinase
Hb: hemoglobin concentration
Ht: hematocrit
IL: interleukin
LmrV: *Lachesis muta rhombeata* venom
PLA_2_: phospholipase A_2_
RBC: red blood cells
SEM: standard error of mean
